# Lack of association between polymorphism rs540782 and primary open angle glaucoma in Saudi patients

**DOI:** 10.1186/s12952-017-0068-1

**Published:** 2017-02-02

**Authors:** Altaf A. Kondkar, Nikhil B. Edward, Hatem Kalantan, Abdullah S. Al-Kharashi, Saleh Altuwaijri, Gamal Mohamed, Tahira Sultan, Taif A. Azad, Khaled K. Abu-Amero

**Affiliations:** 1 Glaucoma Research Chair, Department of Ophthalmology, College of Medicine, King Saud University, Riyadh, 11411 Saudi Arabia; 2 Case Western Reserve University, Ohio, USA; 3 SAAD Research & Development Center, Clinical Research Lab., SAAD Specialist Hospital, P.O. Box 30353, Al Khobar, 31952 Saudi Arabia; 4 Qassim University, Buraydah, Saudi Arabia; 5 Disease Control Strategy Group, Liverpool School of Tropical Medicine, Liverpool, UK; 6 Department of Ophthalmology, College of Medicine, University of Florida, Jacksonville, FL USA; 7 Ophthalmic Genetics Laboratory, Department of Ophthalmology, College of Medicine, King Saud University, P.O. Box 245, Riyadh, 11411 Saudi Arabia

**Keywords:** Middle-east, POAG, rs540782, *ZP4*

## Abstract

**Background:**

To investigate whether polymorphism rs540782 on chromsome 1, in close proximity to the Zona Pellucida Glycoprotein 4 (*ZP4*) gene, is a risk factor for primary open angle glaucoma (POAG).

**Method:**

The study genotyped 92 unrelated POAG cases and 95 control subjects from Saudi Arabia using Taq-Man® assay.

**Results:**

The genotype frequency distribution did not deviate significantly from the Hardy-Weinberg equilibrium (*p* > 0.05). Overall, both the genotype and allele frequencies were not significantly different between cases and controls. The minor ‘C’ allele frequency was 49.4%, which was comparable to the Japanese population and higher than the Indian and Afro-Caribbean populations. Similarly, no significant association was found between genotypes and systemic diseases and health awareness/behavior domain variables. Importantly, glaucoma specific indices, such as intraocular pressure, cup/disc ratio and number of anti-glaucoma medication, also showed no statistically significant effect of genotypes within POAG cases.

**Conclusion:**

Polymorphism rs540782 is not a risk factor for POAG in the Saudi cohort.

## Background

Primary open angle glaucoma (POAG) is largely polygenic in nature with genetically complex and multifactorial inheritance [1]. It is the second most common form of glaucoma in Saudi Arabia and a leading cause of irreversible blindness worldwide [2]. Given the complexity and genetic mutational heterogeneity of POAG, using population-based genome wide association study (GWAS), several investigators have identified a number of variants in multiple loci/genes to be associated with POAG and related quantitative traits that may contribute to the development and/or progression of the disease in various ethnic groups [3].

In 2009, a GWAS study in a group of Japanese POAG patients (*n* = 1575) identified 3 genetic loci consisting of six single nucleotide polymorphisms (SNPs) to be associated with POAG. Of these, 4 intergenic SNPs including rs540782, located on chromosome 1 flanking the Zona Pellucida Glycoprotein 4 (*ZP4*) gene were found to be in strong linkage [4]. *ZP4* gene is involved in functions related to fertilization and pre-implantation development and thus far SNPs and/or mutations in this gene were reported in association with ovarian diseases [5] and POAG [4]. Subsequent studies have failed to replicate this association between rs540782 and POAG in Indian [6], Afro-Caribbean [7] and Japanese [8] populations. Besides, the functional relevance of the SNP or the gene to POAG development is not known.

Despite a high prevalence of POAG in Saudi population the role of genetic polymorphisms in the development of the disease is still unclear [3]. With the aim to identify a genetic link and provide further validation of this POAG-associated variant in a different ethnic group, we investigated whether SNP rs540782 is associated with POAG in the middle-eastern cohort of Saudi Arabia.

## Methods

### Study design and population

We conducted a case-control genetic association study. The study adheres to the tenets of the Declaration of Helsinki and all participants have signed an informed consent. The study was approved by the College of Medicine ethical committee (approval number # 08–657). Saudi patients with a clinically confirmed diagnosis of POAG and a matching group of glaucoma free healthy controls were recruited into the study at King Abdul-Aziz University Hospital (KAUH) in Riyadh, Saudi Arabia. We recruited 92 Saudi POAG patients who satisfied the following strict clinical inclusion criteria for POAG: i) appearance of the disc or retinal nerve fibre layer e.g. thinning or notching of disc rim, progressive changes, nerve fibre layer defect; ii) the presence of characteristic abnormalities in the visual field (e.g. arcuate scotoma, nasal step, paracentral scotoma, generalized depression) in the absence of other causes or explanation; iii) age greater than 20 years at the time of recruitment and iv) open anterior chamber angles bilaterally on gonioscopy. Exclusion criteria included evidence of secondary glaucoma, e.g. pigmentary glaucoma, uveitic, pseudoexfoliation, or any other form of secondary glaucoma, and history of steroid use or ocular trauma. A second group (*n* = 95) of healthy Saudi controls free from glaucoma by examination were recruited. Inclusion criteria for these subjects were age >20 years, normal intraocular pressure (IOP) [IOP < 21 mmHg without any anti-glaucoma medication], open angles on gonioscopy, and normal optic disc on examination.

### DNA preparation

DNA from patients and controls was obtained from peripheral blood (7 mL) collected in EDTA tubes from all participating individuals. Extraction was performed using the illustra blood genomicPrep Mini Spin kit (GE Healthcare, Buckinghamshire, UK) and stored at –20 °C in aliquots until further use. Quantification of extracted DNA was performed using a NanoDrop ND-2000c spectrophotometer (Thermo Scientific, Wilmington, DE, USA).

### Genotyping of rs540782 at Chr.1: 237933586 on GRCh38

Subjects were genotyped to determine the rs540782 polymorphism using the TaqMan® SNP Genotyping Assay (Applied Biosystems Inc., Foster City, CA, USA) on ABI 7500 Real-Time PCR System (Applied Biosystems) as described previously [9]. For detection of rs540782 polymorphism, assay ID: C_8859643_10 was used, Each PCR reaction was performed in a total volume of 25 μL and consisted of 1X TaqMan® Genotyping Master Mix (Applied Biosystems), 1X SNP Genotyping Assay Mix and 20 ng DNA. Each 96-well plate included two no template controls. Real-time PCR was performed on an ABI 7500 using the recommended conditions consisting of incubation at 95 °C for 10 min, followed by 40 cycles, denaturation at 92 °C for 15 s and annealing/ extension at 60 °C for 1 min. The VIC® and 6-carboxy-fluorescein (FAM) fluorescence levels of the PCR products were measured at 60 °C for 1 min. Analysis of fluorescence using the automated 2-color allele discrimination software on ABI 7500 showed clear discrimination of both the genotypes on a two-dimensional graph.

### Statistical analysis

The analysis was done using SPSS version 22 (IBM Inc. Chicago, IIinois, USA). Hardy-Weinberg Equilibrium (HWE) deviation was tested by Pearson’s Chi^2^ test. Odds ratios (OR) were calculated to detect the differences between cases and controls in terms of genotypes and allele frequencies. The Chi^2^ test was used to detect any association between the different genetic profiles (Fishers 2 × 3 Exact test when indicated). Independent samples *t*-test was used to investigate whether there was any significant different between cases and controls in terms of continuous variables. Normality testing of continuous variables was done using Kolmogorov Simrnov test. One-way ANOVA and Kruskal-Wallis Test were used to detect the mean difference across the three genotypes within POAG group. A confidence interval (CI) level was set to 95% where a corresponding *p* value threshold was identified as 0.05 where any output *p* below 0.05 would be interpreted as an indicator of statistical significant.

## Results

### Demographic distribution

As shown in Table 1, the control subjects with a mean age of 57.0 years were found to be slightly younger than the cases with a mean age of 60.7 years, but this was found to be non-significant. The study groups showed a preponderance of male subjects with no statistically significant gender distribution. Besides, except for the family history of glaucoma (*p* = 0.012), both the cases and control groups were found to be similar for systemic co-morbidities, smoking habit and glaucoma specific clinical indices.

**Table 1:** Clinical and demographic characteristics POAG cases and controls

Variables	Controls (*n* = 95) No. (%)	Cases (*n* = 92) No. (%)	*p* value^a^
Demographic Characteristics			
Age in years, Mean (±SD)	57.2 (13.5)	60.7 (12.3)	0.065*
Male	69 (72.6)	56 (60.8)	0.120
Female	26 (27.3)	36 (39.1)	-
Systemic Diseases			
Diabetes mellitus	45 (47.3)	49 (53.2)	0.465
Coronary artery disease	6 (6.3)	7 (7.6)	0.780
Hypertension	44 (46.3)	48 (52.1)	0.465
Hypercholesterolemia	9 (9.4)	15 (16.3)	0.192
Health Awareness/Behavior			
Family history of glaucoma	4 (4.2)	14 (15.2)	0.012
Smoking	41 (43.1)	34 (36.9)	0.456

^a^Chi^2^ test

*t-test

### Genotype and allele frequency

The SNP did not deviate significantly from the HWE (*p* > 0.05) in both the cases and control groups. Basic allelic testing showed that the allele frequency distribution was non-significant between the two groups (OR = 1.11; 95% CI = 0.74–1.67; *p* = 0.606). The minor “C” allele frequency was found to be 0.46 and 0.49 among POAG cases and controls, respectively. The wild type (G/G) genotype was detected in 26.3% controls as compared to 23.9% in cases. Besides, the heterozygous C/G genotypes was observed in 48.4% controls as compared to 58.7% cases and the homozygous mutant C/C genotype in 25.2% and 17.4% of controls and cases, respectively. However, association testing for SNP rs540782 between cases and controls revealed no significant genotype distribution under additive (Chi^2^ = 2.38, df = 2, *p* = 0.30), dominant (*p* = 0.70) and recessive models (*p* = 0.69) (Table 2).

**Table 2:** Association results of SNP rs540782 with genotype and allele frequency distribution in POAG and controls

SNP (Gene)	rs540782 (*ZP4*)				
Allelic analysis	Controls (*n* = 95) No. (%)	POAG (*n* = 92) No. (%)	Odds ratio	95% confidence interval	*p* value^a^
G	96 (50.5)	98 (53.2)	1	Reference	-
C*	94 (49.4)	86 (46.7)	1.11	0.74–1.67	0.60
HWE P	0.76	0.09	-	-	-
Genotype and Model analysis					
G/G	25 (26.3)	22 (23.9)	1	Reference	-
C/G	46 (48.4)	54 (58.7)	0.75	0.37–1.50	0.48
C/C	24 (25.2)	16 (17.4)	1.32	0.56–3.09	0.66
Additive (Trend)	-	-	-	-	0.30^§^
Dominant	-	-	0.88	0.45–1.70	0.70
Recessive	-	-	1.16	0.54–2.0	0.69

^a^Chi^2^ test

*Risk variant

*HWE P* Hardy-Weinberg equilibrium *p* value

^§^Fisher exact test

### Genotype effect on demographic and clinical parameters in POAG

Age (*p* = 0.713) and gender (*p* = 0.617) did not show significant difference between genotypes. Similarly, none of the systemic diseases and health awareness/behavior domain variables showed any significant difference. Importantly, glaucoma specific indices such as IOP, cup/disc ratio and number of anti-glaucoma medication also showed no statistically significant difference between the genotype groups (Table 3).

**Table 3:** Effect of genotypes on demographic and clinical characteristics within PAOG cases

Characteristics	Genotypes			*p* value^a^
	G/G (*n* = 22) No. (%)	G/C (*n* = 54) No. (%)	C/C (*n* = 16) No. (%)	
Demographic				
Age in years, Mean (SD)	59.0 (13.5)	61.5 (12.0)	60.2 (12.0)	0.713*
Male	14 (25.0)	34 (60.7)	8 (14.2)	0.617
Female	8 (22.2)	20 (55.5)	8 (22.2)	-
Medical history				
Family history of glaucoma	4 (28.5)	9 (64.2)	1 (7.1)	0.613
Diabetes mellitus	13 (26.5)	28 (57.1)	8 (16.3)	0.813
Smoking	11 (32.3)	17 (50.0)	6 (17.6)	0.316
Hypertension	10 (20.8)	30 (62.5)	8 (16.6)	0.713
Coronary artery disease	2 (28.5)	4 (57.1)	1 (14.2)	0.944
Hypercholesterolemia	5 (33.3)	8 (53.3)	2 (13.3)	0.630
Glaucoma indices				
Intraocular pressure in mmHg, Mean (SD)	33.2 (6.2)	34.7 (8.6)	31.9 (5.7)	0.474**
Cup/disc ratio	0.68 (0.19)	0.74 (0.15)	0.64 (0.24)	0.243**
No. of anti-glaucoma medications	2.8 (0.6)	2.8 (0.8)	2.5 (0.6)	0.122**

^a^Chi^2^ test

*One-way ANOVA

**Kruskal Wallis test

Besides, logistic regression was performed to ascertain the effects of age, gender and genotype on the likelihood of having POAG. However, none of the variables could significantly explain the likelihood of POAG in this cohort (Table 4).

**Table 4:** Effect of age, sex and genotype on disease outcome by logistic regression analysis

Variables	Odds ratio	95% confidence interval	*p* value
Age	1.02	0.99–1.04	0.103
Sex^a^	0.59	0.32–1.11	0.104
Genotype^b^			0.303
C/G	1.236	0.608–2.51	0.558
C/C	0.727	0.306–1.73	0.471

^a^Female as reference

^b^G/G as reference

## Discussion

This study investigated the association between SNP rs540782, which is in close proximity to *ZP4* gene on chromosome 1, and POAG in the Saudi patients. Previously this SNP was reported to be associated with POAG in a group of Japanese patients (*p* = 0.00006, OR = 1.34, 95% CI = 1.16–1.54) [4]. However, the authors did not offer any explanation on how SNP rs540782 contributes to POAG-pathogenesis. Subsequent studies have failed to establish a link between this SNP and POAG [6–8]. In our study, the genotype and allele frequencies detected in POAG patients were comparable to those in controls and thus were insignificant. This is similar to previous investigations which reported no association between this SNP and POAG or any of its clinical indices [6–8]. The minor allele (C) frequency detected in the Saudi population (controls) was 49.4%, which is similar to the Japanese population’s 49.5% [8], but higher than that for the Indian (35.6%) [6] and Afro-Caribbean (29.9%) populations [7].

Association analysis of the genotype effect on clinical parameters also did not provide any significant link. None of the systemic diseases and health awareness/behavior domain variables such as family history of glaucoma and smoking showed any significant difference. Importantly, glaucoma specific indices such as IOP, cup/disc ratio and number of anti-glaucoma medication also showed no statistically significant difference between the genotype groups. Therefore, this SNP, independently or in relation to other clinical indices, does not have any effect on POAG development.

## Conclusion

This study failed to detect any direct link between genotype and allele frequency of SNP rs540782 and POAG or its related clinical indices, such as IOP and cup/disc ratio, indicating that this polymorphism is not a risk factor for POAG in the Saudi cohort.

## Abbreviations

CI: Confidence interval
GWAS: Genome-wide association study
HWE: Hardy-Weinberg Equilibrium
IOP: Intraocular pressure
OR: Odds ratio
POAG: Primary open angle glaucoma
SNP: Single nucleotide polymorphism
*ZP4*: Zona Pellucida Glycoprotein 4
